# Supplementary material for: MetaRibo-Seq measures translation in microbiomes
Source: Nat Commun. 2020 Jun 29;11:3268. doi: 10.1038/s41467-020-17081-z (PMC7324362; doi:10.1038/s41467-020-17081-z)
Supplement: Supplementary file 10 — Supplementary Data 7 [file 41467_2020_17081_MOESM10_ESM.zip › File2/Confidence_High_Taxonomy/138791_out.krona.html]

Javascript must be enabled to view this page.

members
magnitude
magnitudeUnassigned
count
unassigned
taxon
rank

138791\_out

10

10
2
superkingdom

phylum
256845
10

1313211
10
class

species

SRS018836\_contig\_number\_17789SRS024009\_contig\_number\_contig-100\_12198.12198SRS055017\_contig\_number\_contig-100\_2593.104816SRS075078\_contig\_number\_contig-100\_256.165879SRS077392\_contig\_number\_contig-100\_19.229082SRS078242\_contig\_number\_31272SRS1041145\_contig\_number\_25118SRS142599\_contig\_number\_26236SRS143895\_contig\_number\_25879SRS144183\_contig\_number\_16454
10
2053569
